# The risk of intra-cranial haemorrhage in those presenting late to the ED following a head injury: a systematic review

**DOI:** 10.1186/s13643-015-0154-8

**Published:** 2015-11-18

**Authors:** Carl Marincowitz, Christopher M. Smith, William Townend

**Affiliations:** Emergency Department, Hull Royal Infirmary, Anlaby Road, Hull, HU3 2JZ UK

**Keywords:** Head injury, Delayed presentation, Emergency department, Intra-cranial haemorrhage, Intra-cranial bleed

## Abstract

**Background:**

Head injury represents an extremely common presentation to emergency departments (ED), but not all patients present immediately after injury. There is evidence that clinical deterioration following head injury will usually occur within 24 h. It is unclear whether this means that head injury patients that present in a delayed manner, especially after 24 h, have a lower prevalence of significant traumatic injuries including intra-cranial haemorrhages.

**Methods:**

A systematic review protocol was designed with the aim of systematically identifying and evaluating studies in delayed ED presentation head injury populations in order to establish whether the prevalence of significant intra-cranial injury was affected by delay in presentation. Two independent researchers assessed retrieved studies for inclusion against pre-determined inclusion criteria. Studies had to be conducted in ED head injury populations presenting in a delayed manner, and report a measure of prevalence of traumatic CT abnormality as an outcome.

**Results:**

Three studies were eligible for inclusion. They were all of poor methodological quality, and heterogeneity prevented meta-analysis. The reported prevalence of traumatic intra-cranial injury on CT was between 2.2 and 6.3 %. This is generally lower than reported in the literature for non-delayed presentation head injury populations.

**Conclusions:**

Available evidence suggests that head injury patients who present in a delayed fashion to the ED may have lower rates of intra-cranial injury compared to non-delayed head injury patients. However, the evidence is sparse and it is of too low quality to guide clinical practice. Further research is required to help the clinical risk assessment of this group.

**Trial registration:**

PROSPERO: CRD42015016135

## Background

There are approximately 1.4 million attendances to emergency departments (ED) in England and Wales following head injury each year [1]. Ninety-five percent of these attendances are patients with minor/mild head injuries, as defined by a Glasgow Coma Scale (GCS) score of 13, 14 or 15 [1, 2]. Research has been directed at differentiating patients with minor/mild head injury into two groups: those who are at sufficiently low risk to be discharged on the basis of clinical history and examination alone and those who require a computed tomography (CT) scan of the head to rule out serious intra-cranial pathology. In the UK, National Institute for Health and Clinical Excellence (NICE) guidelines are used to facilitate this risk assessment. These are, in turn, based upon the Canadian CT head rules (CCHR) [3]. The CCHR, however, were derived from a population of patients presenting within 24 h. Both the NICE guidelines and CCHR have only been validated in populations of patients presenting within 24 h [1, 4–9].

Not all patients present to the ED immediately after sustaining a head injury, particularly if they fall into the minor/mild head injury group. Some present after 24 h [10]. The paucity of research in this area means that estimating the size of this population is difficult. However, one study found that a third of a cohort of minor head injury patients presenting after 4 h of injury sustained their injury more than 24 h previously [10]. Local audit data (Hull Royal Infirmary, Nov 2011–May 2012) indicates that approximately 15 % of CT head scans for adult head trauma were requested for patients who presented after 24 h of injury (unpublished data). There is evidence that patients with a minor head injury and intra-cranial haemorrhage who deteriorate clinically do so within 24 h [11, 12], with the majority deteriorating within 8 h [12]. This suggests that patients presenting after this time, with signs and symptoms suggesting of minor/mild head injury, may be a selected sub-population at a lower risk of significant intracranial pathology.

However, there are case reports in the literature of patients with delayed onset intra-cranial haemorrhage following a head injury [13, 14]. The occurrence appears more likely in anti-coagulated patients [15, 16]. Moreover, the Australian New South Wales (NSW) Health Guidelines identifies patients who present in a delayed fashion as a potentially higher-risk group due to the persistence or worsening of symptoms [17]. These were the only national or regional guidelines regarding the management of patients with a delayed presentation of head injury to the ED that we could find. The NSW guidelines concede that its advice regarding delayed presentation head injury patients has a poor evidence base.

Furthermore, patients who re-present to the ED after an initial acute presentation following head injury have been identified as a high-risk group [18]. We postulate that this is because patients are re-presenting following an observable change (either by them or those caring for them) in their clinical state, and/or because they are acting on direct advice given to them at their original ED visit about when they should re-present.

It is currently unclear whether the time of presentation to the ED after head injury correlates with an altered likelihood of intra-cranial pathology. This has implications when applying existing guidelines to the risk assessment of patients with minor/mild head injury who have a delayed presentation. If deterioration from neurosurgical pathology occurs within a fixed acute time frame, patients presenting after this should re-present a more benign sub-population with a lower prevalence of identified significant intra-cranial injuries. Alternatively, if they are a self-selecting high-risk group presenting due to worsening or persistence of symptoms with underlying slower developing pathology or secondary intra-cranial bleeds, the prevalence of injuries should be correspondingly higher.

This study aims to systematically identify and evaluate studies that measure the estimated prevalence of traumatic intra-cranial injury in delayed presentation head injury populations.

## Methods

The PRISMA systematic review checklist was used in the formulation of the systematic review methodology [19]. The systematic review is registered on the PROSPERO prospective register of systematic reviews (http://www.crd.york.ac.uk/PROSPERO/display_record.asp?ID=CRD42015016135). The protocol is available there.

### Research ethics

No specific ethical approval was required for this study as it is a systematic review.

### Search strategy

Relevant terms related to delayed diagnosis and intracranial pathology were identified after reviewing both the PubMed PubReMiner service (http://hgserver2.amc.nl/cgi-bin/miner/miner2.cgi) and Medical Subject Headings (MESH—via the US National Library for Medicine MESH browser at http://www.nlm.nih.gov/mesh/MBrowser.html). From this, an electronic search strategy was devised. Articles of potential interest were identified from searches in MEDLINE (Ovid MEDLINE(R) In-Process & Other Non-Indexed Citations and Ovid MEDLINE(R) 1946 to Present) and EMBASE (1974 to 2015 January 23) (Wolter Kluwers Health at http://ovidsp.uk.ovid.com/sp-3.13.1a/ovidweb.cgi). The full search strategy and the date searches undertaken are attached in the Appendix.

Further articles were identified via a bibliography search of full-text articles retrieved from the electronic searches and by using the ‘related articles’ features of PubMed and Google Scholar. Further free-text searches of Google Scholar, PubMed and NICE Evidence Search (https://www.evidence.nhs.uk/) were also undertaken. Free-text searches yielded information about NICE [1], NSW [17] and Scottish Intercollegiate Guidelines Network (SIGN) Head Injury guidelines [20], whose bibliographies were again interrogated for studies of potential relevance.

### Inclusion criteria

All study types except isolated case studies were considered for inclusion. Studies had to be conducted in ED populations who had sustained a head injury, presenting in a delayed manner. After a scoping review suggesting a paucity of articles, no fixed time definition was used for what constituted a delay. Both adult and paediatric populations were considered. Studies had to include enumeration of significant clinical intracranial traumatic pathology on CT head scan as an outcome measure.

### Study selection

Articles were considered for inclusion through a title and abstract review of papers identified from the electronic searches and by review of bibliographies and related articles by two independent reviewers (CM and CMS). A list of potentially relevant studies was identified, and the full-texts of these studies were obtained and reviewed for final inclusion. Any uncertainty or disagreement was resolved after discussion between the two reviewers.

### Assessment of methodological quality

It was thought that the search strategy would identify cohort studies. The reviewers planned to assess their methodological quality by utilising the Newcastle Ottawa Scale [21]. However, the studies identified were observational studies with no comparators. Therefore, a descriptive assessment of bias for each study was undertaken and this was informed by the Cochrane handbook [22].

### Data extraction and synthesis

Data was reviewed and variables relating to study population, design, outcome measures and results were extracted. An assessment of methodological quality was made.

## Results

**Fig. 1:**
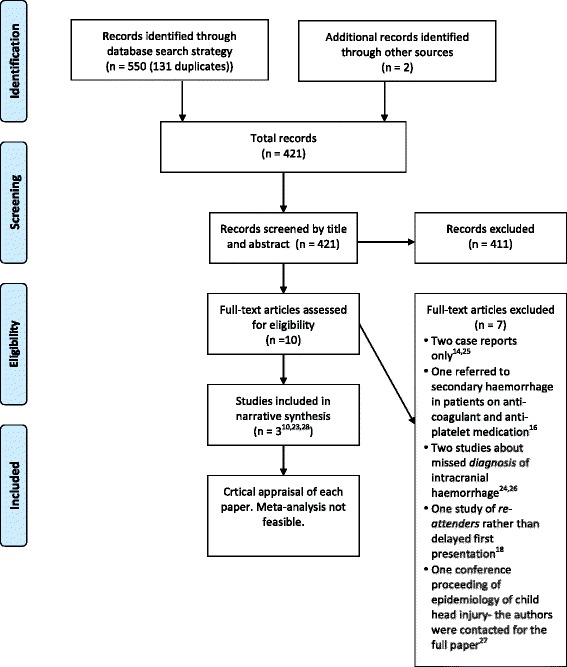
The PRISMA flow chart. This flow chart is modified from http://www.prisma-statement.org and it shows the study identification and selection process

The MEDLINE and EMBASE search returned 419 potentially relevant articles. Eight were selected for full-text review [10, 14, 16, 23–27], and two met the criteria for inclusion into the systematic review [10, 23]. Other search strategies yielded two additional articles of potential interest [18, 28]. One of these (identified from bibliography search) was included in the systematic review [28]. This paper was in abstract form only: we received no reply to the multiple attempts made to contact the authors. There was no record of this information being published as a full paper elsewhere. The information is presented in Fig. 1, including the characteristics of articles excluded after full-text review.

### Study characteristics and quality assessment

The included studies are summarised in Table 1. One prospective observational English study [10], one retrospective observational US study [23] and a US case series (abstract only) [28] were identified that defined delay in presentations as 4 [10], 12 [23] and 4 h [28], respectively. The populations in which studies were conducted also differed: Barrow et al. [10] studied patients aged 17 years or older presenting with a GCS of 14 or 15; Hemphill et al. [23] studied patients of any age presenting with GCS 15; and Borczuk et al. [28] included patients aged 16 years or older but did not state the presenting GCS, and only included patients who had undergone a CT head scan. The main outcome in all three studies was defined as any traumatic abnormality on CT head scan. This rate was found to be 2.21 % [10], 3.1 % [23] and 6.3 % [28]. Meta-analysis of the data was not possible due to the low number and heterogeneity of identified studies.

**Table 1:** Characteristics of included studies

Reference	Population	Study design	Outcome measures	Results	Quality appraisal
Barrow et al. [10]	Inclusion criteria: • Age ≥ 17 years • GCS 14 or 15 at presentation • Presenting to ED >4 h after injury Exclusions: • Patients with only facial injuries Single-site: large central-east London teaching hospital 1st Jan. 2008–10th May 2009	Prospective observational study	‘Positive CT’: any traumatic finding related to presenting injury	Detailed patient demographics not reported 497 patients included: • 193 presented 4–12 h • 140 presented 12–24 h • 62 presented 24–28 h • 58 presented 24–168 h • 44 presented ≥ 1 week 147/497 (29.6 %) had CT head; 64/147 presented 4–12 h, 50/147 12–24 h, 11/147 24–48 h, 21/147 48 h–1 week, 1/147 > 1 week 11/497 (2.21 %) positive CT scans; 1/11 presented 4–12 h, 3/11 12–24 h, 4/11 24–48 h, 3/11 48–168 h 4/497 (0.80 %) had neurosurgery; 3/4 (75 %) presented 12–48 h after injury, 1/4 (25 %) 48–168 h 1/497 died (0.20 %)—time since presentation not reported 69/497 (13.9 %) contactable at 2 weeks; 11/69 (15.9 %) symptomatic Lower rates of intra-cranial injury compared to previous studies Similar rates of CT and neurosurgical intervention compared to previous studies Statistically significant predictors of intra-cranial injury: LOC, coagulopathy, evidence of injury above the clavicles, open or depressed skull fracture and acute alcohol/drug use	Prospective and contemporaneous review of notes—likely that most eligible cases were identified and included
		NICE guidelines used for triage patients to CT head and discharge	2–4 week telephone interview follow-up for further treatment/deterioration		4 h is not a long delay
		Data collection: Daily identification of cases from search of paper records and review of computerised discharges	Identification of clinical risk factors predictive of intra-cranial injury		No control or comparison group
					Sampling biases: small numbers, young population, >50 % from Indian subcontinent
					Small absolute rates of pathology, therefore prone to outlier bias
					Very high loss to follow-up
Hemphill et al. [23]	Inclusion criteria: • Any age • GCS 15 at presentation • Presenting to ED >12 h after injury • Re-attenders included Exclusions: • None stated Dual-site: academic Level I Trauma Centres (San Antonio, USA) Jan.–Dec. 1996	Retrospective chart review Searched 85,000 ED charts	‘Significant delayed injury’. Defined as ‘abnormal CT results such as: intracerebral bleeding, skull fracture, or subdural or epidural haematoma’	2900 patients with head injury 194 (6.69 %) presented >12 h: • 112/194 (56.9 %) female • 34 ± 24 years (mean ± SD) • 21/194 (10.8 %) re-attenders 101/194 (52.1 %) patients had CT head; 9/21 (42.9 %) of re-attenders had CT head scan 6/194 (3.1 %) patients had abnormal CT scans: • 2 infants (aged 1 and 5 months) • 4 adults (29F, 46F, 60M, 74M) • Note: one patient (74M) presented GCS 3 with large DSH at 25 h *after* normal CT acutely after injury—required neurosurgery and died 1 patient re-presented at 3 months with headache: chronic SDH—did not originally have CT head Mean time to presentation: • Overall: 73 ± 105 h • If abnormal CT: 29.3 ± 10.7 h	Retrospective review—data may be missing
			Comparisons between patients with/without CT		Exclusions not stated
			Comparisons between hospitals		No formal follow-up of patients who did not have CT head scans
					Sampling bias: small numbers
					Small absolute rates of pathology, therefore prone to outlier bias
					Includes re-attenders: a distinct and possibly higher-risk group than delayed (first-time) presenters
Borczuk et al. [28] Abstract only	Inclusion criteria: • Age > 16 years • Presenting to ED >24 h after injury • Blunt head injury • Had CT head Exclusions: • Not stated Single-site: MA, USA Conducted ‘over a 2-year period’ (dates not reported)	Case series	Any abnormality on CT head	206 consecutive patients identified GCS on presentation not stated 13/206 (6.3 %) had abnormality on CT head No patient required neurosurgery Time to presentation (mean ± SD): • Positive CT findings: 5.03 ± 6.52 days • Negative CT findings: 5.79 ± 7.09 days Positive CT findings more likely if LOC or amnesia of the event reported	Case series—does not state which patients were excluded or how
					Sampling biases: small numbers, only those who had CT head after injury were included
					GCS/other indicators of injury severity not discussed
					Abstract only—unable to contact authors for further information
					‘Abnormality’ on CT not defined or explained further

#### Key limitations of included studies

None of the studies measured the prevalence of intracranial abnormality in patients who presented acutely after injury, so it was not possible to make any contemporaneous comparison between rates of intracranial abnormality between delayed and non-delayed ED presentation head injury patients.

Barrow et al. [10] is the only recent study. The very high loss to follow-up of patients who did not have CT scans, and the low overall rate of pathology, makes the study susceptible to attrition bias.

The paper by Hemphill et al. [23] was published in 1999. It pre-dates current guidelines for CT head scanning in head injury and has a relatively high CT head scan rate. This reflects practice at this time in the USA, but makes it difficult to generalise the findings to current practice. No formal attempt was made to follow up patients who had not had scans. If they had not re-attended to the same ED, it is possible that intra-cranial pathology was under-reported. This, coupled with the low rate of pathology, again makes the study highly susceptible to attrition and outlier bias.

Borczuk et al. [28] present an abstract of a case series. It includes no exclusion criteria or attempt to measure the number of patients who presented after 24 h and did not have scans. Attempts to contact the authors for further information were not successful. It is difficult to draw any meaningful conclusions about this study that are relevant to current practice. Indeed, the small absolute amount of pathology and peculiarities of the single centres where all three studies were conducted makes them liable to the affects of outlier and sampling biases.

## Discussion

There is a paucity of research about patients with head injury who present to the ED in a delayed manner after head injury. This systematic review confirms that there are a few studies in this area and that high-quality data is lacking.

A large systematic review found the median prevalence of intracranial injury in patients with minor/mild head injury to be 7.2 % [7]. This is almost exclusively based on studies conducted in populations presenting within 24 h. The prevalence of intra-cranial pathology in the included studies here [10, 23, 28] was lower, although direct comparisons between delayed and early presentation groups were not undertaken in these studies. We also cannot determine whether a potential reduction in intra-cranial pathology in those who have delayed presentation to the ED after head injury would translate to lower rates of neurosurgical intervention or death. Barrow et al. [10] reported similar rates of neurosurgery to previous studies in patients presenting early after head injury [4, 29], whereas Borczuk et al. [28] reported no patients requiring neurosurgical intervention in a pre-selected group who all underwent a CT head scan.

Current NICE guidelines are based on research in populations presenting within 24 h. The authors have observed a degree of variation in clinical practice in the cranial CT imaging of patients presenting after this. Some clinicians appear to have a low threshold to image any symptomatic patients that do not present immediately after injury. Contrastingly, some clinicians do not image this group despite the presence of NICE guideline indications. There appears to be some consensus that patients taking warfarin or with severe headaches that present after a delay following a head injury should undergo a CT head scan. Head injury patients that present in a delayed manner may have a distinct risk profile. Application of existing guidelines to this group may risk over-investigation or, conversely, risk missing important intra-cranial injuries.

## Conclusions

The relatively sparse data available suggest that there is a lower incidence of traumatic intra-cranial pathology for those presenting 24 h after injury, but this is insufficient to guide clinical practice. Further research is required to characterise the delayed presentation ED head injury population and establish the prevalence and associated risk factors of significant intra-cranial injury in this group.

## Appendix

**Table 2:** EMBASE search undertaken 23/1/2015 and Medline search undertaken 29/1/2015

	MEDLINE	EMBASE
1. exp Delayed Diagnosis/	2599	6141
2. delayed presentation.mp. [mp=title, abstract, original title, name of substance word, subject heading word, keyword heading word, protocol supplementary concept word, rare disease supplementary concept word, unique identifier]	1306	1627
3. late presentation.mp. [mp=title, abstract, original title, name of substance word, subject heading word, keyword heading word, protocol supplementary concept word, rare disease supplementary concept word, unique identifier]	1653	2246
4. exp Cerebral Hemorrhage/ or exp Intracranial Hemorrhages/	56224	91897
5. (intracranial haemorrhage or intracranial hemorrhage or intracerebral haemorrhage or intracerebral hemorrhage).mp. [mp=title, abstract, original title, name of substance word, subject heading word, keyword heading word, protocol supplementary concept word, rare disease supplementary concept word, unique identifier]	15271	22369
6. (skull fracture or brain contusion or basal skull fracture).mp. [mp=title, abstract, original title, name of substance word, subject heading word, keyword heading word, protocol supplementary concept word, rare disease supplementary concept word, unique identifier]	1899	11318
7. exp Craniocerebral Trauma/	124238	225415
8. (head injur* or brain injur*).mp. [mp=title, abstract, original title, name of substance word, subject heading word, keyword heading word, protocol supplementary concept word, rare disease supplementary concept word, unique identifier]	76828	150849
9. 1 or 2 or 3	5352	9752
10. 4 or 5 or 6 or 7 or 8	192518	322486
11. 9 and 10	168	382

## Abbreviations

CCHR: Canadian CT head rule
CT: computed tomography
ED: emergency department
GCS: Glasgow Coma Scale
NICE: National Institute for Health and Clinical Excellence
NSW: New South Wales
SIGN: Scottish Intercollegiate Guidelines Network
